# Oral Probiotics Alter Healthy Feline Respiratory Microbiota

**DOI:** 10.3389/fmicb.2017.01287

**Published:** 2017-07-11

**Authors:** Aida I. Vientós-Plotts, Aaron C. Ericsson, Hansjorg Rindt, Carol R. Reinero

**Affiliations:** ^1^College of Veterinary Medicine, University of Missouri Columbia, MO, United States; ^2^Department of Veterinary Medicine and Surgery, College of Veterinary Medicine, University of Missouri Columbia, MO, United States; ^3^Comparative Internal Medicine Laboratory, University of Missouri Columbia, MO, United States; ^4^University of Missouri Metagenomics Center, University of Missouri Columbia, MO, United States; ^5^Department of Veterinary Pathobiology, College of Veterinary Medicine, University of Missouri Columbia, MO, United States

**Keywords:** respiratory, operational taxonomic unit (OTU), 16S rRNA gene, probiotics, richness, abundance, diversity, translational research

## Abstract

Probiotics have been advocated as a novel therapeutic approach to respiratory disease, but knowledge of how oral administration of probiotics influences the respiratory microbiota is needed. Using 16S rRNA amplicon sequencing of bacterial DNA our objective was to determine whether oral probiotics changed the composition of the upper and lower airway, rectal, and blood microbiota. We hypothesized that oral probiotics would modulate the respiratory microbiota in healthy cats, demonstrated by the detection and/or increased relative abundance of the probiotic bacterial species and altered composition of the microbial population in the respiratory tract. Six healthy young research cats had oropharyngeal (OP), bronchoalveolar lavage fluid (BALF), rectal, and blood samples collected at baseline and 4 weeks after receiving oral probiotics. 16S rRNA gene amplicon libraries were sequenced, and coverage, richness, and relative abundance of representative operational taxonomic units (OTUs) were determined. Hierarchical and principal component analyses (PCA) demonstrated relatedness of samples. Mean microbial richness significantly increased only in the upper and lower airways. The number of probiotic OTUs (out of 5 total) that significantly increased in relative abundance vs. baseline was 5 in OP, 3 in BAL and 2 in feces. Using hierarchical clustering, BALF and blood samples grouped together after probiotic administration, and PERMANOVA supported that these two sites underwent significant changes in microbial composition. PERMANOVA revealed that OP and rectal samples had microbial population compositions that did not significantly change. These findings were visualized via PCA, which revealed distinct microbiomes in each site; samples clustered more tightly at baseline and had more variation after probiotic administration. This is the first study describing the effect of oral probiotics on the respiratory microbiota via detection of probiotic species in the airways. Finding bacterial species present in the oral probiotics in the upper and lower airways provides pilot data suggesting that oral probiotics could serve as a tool to target dysbiosis occurring in inflammatory airway diseases such as feline asthma, a disease in which cats serve as an important comparative and translational model for humans.

## Introduction

Prior to the advent of culture-independent microbiological techniques, the lower airways were considered sterile, and the primary role of bacteria in respiratory disorders was assumed to be as pathogens leading to development, persistence, exacerbation, and/or progression of respiratory diseases (Beigelman et al., 2014). However, recent studies using sequencing of microbial 16S rRNA amplicons (Sidiq et al., 2016) have demonstrated that healthy humans (Charlson et al., 2012), dogs (Ericsson et al., 2016), and cats (Vientos-Plotts et al., 2017) harbor rich, complex commensal microbial populations in the respiratory tract. There is increasing evidence that commensal microbes play a critical role in the maturation, education, and function of the immune system and, in particular, regulation of inflammatory responses. Additionally, dysbiosis, or deviations from the healthy microbiota, is associated with overgrowth of pathogens, decreased biodiversity, and stimulation of the host immune system that contributes to disease development (Gollwitzer and Marsland, 2014; Fujimura and Lynch, 2015). Dysbiosis has been documented in a variety of human respiratory diseases such as asthma (Huang, 2015; Ling et al., 2016), chronic obstructive pulmonary disease (COPD) (Gomez and Chanez, 2016; Huang et al., 2017), cystic fibrosis (Hogan et al., 2016; Madan, 2016), and respiratory tract infections (Pirrone et al., 2016; Yin et al., 2017).

Dietary supplementation in the form of probiotics, live microorganisms that provide a health benefit to the host when administered in adequate amounts (Guarner et al., 2012), beneficially alter the gut-lung axis (Forsythe, 2011; Kalyuzhin et al., 2016). *Lactobacillus, Streptococcus*, and *Bifidobacterium* species are some of the most common organisms considered probiotics. The specific mechanisms of action for health benefits of different probiotic strains are poorly understood. Probiotic strains may exert their effects on dysbiosis directly by changing the composition of the host microbiome or indirectly by interacting with the host through the common mucosal immune system (CMIS) (Neish, 2014; Thaiss et al., 2016). The CMIS is a unique branch of the immune system shared by mucosal sites; importantly, it enables cross-talk between the gastrointestinal (GI) and respiratory tracts (Budden et al., 2016; Ghaisas et al., 2016). In humans, orally administered probiotics modulate immune responses in the lung, promoting a tolerogenic environment of benefit in allergic disease (Rautava and Isolauri, 2002).

To our knowledge, no studies have evaluated the effect of oral probiotics on the airway microbiota of healthy cats, a species of importance for translational models of respiratory disease including allergic asthma (Norris Reinero et al., 2004; Reinero et al., 2009). The objective of the current study was to determine the effect of oral probiotics on the composition of the upper and lower airway, rectal, and blood microbiota. We hypothesized that probiotics would modulate the respiratory microbiota in healthy cats, demonstrated by the detection and/or increased relative abundance of the probiotic bacterial species and altered composition of the commensal microbial population in the respiratory tract after treatment. Furthermore, we postulated that alterations in rectal and blood microbiota might provide clues for mucosal or hematogenous transport of probiotic species.

## Materials and methods

### Ethics statement

All studies were performed in accordance with the Guide for the Use and Care of Laboratory Animals, and were approved by the University of Missouri Institutional Animal Care and Use Committee (MU IACUC protocol #7891).

### Cats

Cats were bred from a colony (Comparative Internal Medicine Laboratory, University of Missouri, Columbia, MO), were all sexually intact and were aged <1 year (31–41 weeks by end of study). Cats were housed by sex with 2 males and 4 females housed in separate, large runs with elevated platforms for climbing and enrichment toys. Cats were transitioned to a commercial dry diet formulated for growth (Purina growth formula, St. Louis, MO) at 4 weeks of age, and remained on this diet for the duration of the study. Access to food and clean drinking water were provided *ad libitum*. In order to reduce risk of aspiration, cats were fasted for at least 12 h prior to anesthesia for sample collection.

Cats were determined to be healthy by absence of respiratory clinical signs, a normal physical examination by a board-certified veterinary internal medicine specialist and lack of cytologic evidence of infection or inflammation from bronchoalveolar lavage fluid (BALF) samples. Euthanasia was not an endpoint of the study; all cats were subsequently adopted into private homes.

### Probiotic administration protocol

Cats were administered one capsule of VSL#3 probiotic mixed in their food twice daily (225 × 10^9^ CFU/day), for 4 weeks. Cats were individually fed a portion of their meal with the probiotic to ensure complete consumption before receiving the remainder of the meal.

### Sample collection

Rectal swabs, oropharyngeal (OP) swabs, BALF and blood were collected at the beginning of the study (baseline), and immediately after 4 weeks of receiving probiotics. Samples were collected as previously described (Vientos-Plotts et al., 2017). Briefly, cats were anesthetized and a sterile cotton-tip swab was inserted rectally, while avoiding contact with the perianal area. After induction, but prior to intubation, a second moistened sterile swab was used to rub the caudodorsal aspect of the oropharynx, while avoiding contact with the rest of the oral cavity. The OP and rectal swabs were each added to 5 mL of sterile saline. Cats were carefully intubated using a sterile 3.5–4 French endotracheal tube. To collect BALF, a 20 mL aliquot of sterile saline was instilled via a sterile 8 French red rubber catheter that was threaded through the endotracheal tube. Four milliliters of whole blood were obtained by jugular venipuncture (site shaved of fur and prepared with ethanol) into sterile tubes with the anticoagulant EDTA. Immediately after collection, all samples were placed on ice and transported to the laboratory.

Promptly after collection, rectal, OP, BALF, and blood samples were centrifuged to pellet bacterial cells. Red blood cells (RBCs) were lysed prior to centrifugation of the blood samples. To lyse RBCs, 3 mL of blood was mixed with 27 mL sterile water and incubated at room temperature for 1 min before adding 3.3 mL of 10 × PBS and centrifuging for 20 min at 2,000 × g. In all cases, supernatant was discarded and pellets were resuspended in 800 μL lysis buffer adapted from Yu et al. (4% sodium dodecyl sulfate, 50 mM EDTA, 500 mM NaCl, and 50 mM Tris-HCl pH 8.0) (Yu and Morrison, 2004). All samples were banked at −80°C until the end of the study, and DNA was extracted as a single batch.

### DNA extraction, 16S rRNA library preparation, sequencing, and informatics

DNA from feces, OP, and BALF, and blood was extracted using the column method as previously described (Ericsson et al., 2016; Vientos-Plotts et al., 2017). Library construction and sequencing was completed at the University of Missouri DNA Core facility as previously described (Ericsson et al., 2016). Briefly, libraries were generated using single-indexed universal primers (U515F/806R) flanked by Illumina adapter sequences and PCR was performed using the following parameters: 98°C^(3:00)^ + [98°C^(0:15)^ + 50°C^(0:30)^ + 72°C^(0:30)^] × 25 cycles + 72°C^(7:00)^. Amplicons were pooled for sequencing on the Illumina MiSeq platform using the V2 chemistry and 2 × 250 paired-end reads. No samples returning less than 200 reads for BALF or blood, less than 1,350 for OP swabs, or less than 10,000 reads for rectal swabs were included. Assembly, filtering, binning, and annotation of DNA sequences was performed at the MU Informatics Research Core Facility as previously reported (Vientos-Plotts et al., 2017). Briefly, DNA contigs were assembled using FLASH software (Magoc and Salzberg, 2011) and culled if found to be short after trimming for base quality below 31. Qiime v1.8 (Kuczynski et al., 2011) was used to perform *de novo* and reference-based chimera detection and removal, and remaining contigs were assigned to operational taxonomic units (OTUs) via *de novo* OTU clustering using BLAST (Altschul et al., 1997) against the Greengenes database (DeSantis et al., 2006) of 16S rRNA sequences and taxonomy. Principal coordinate analysis of Bray-Curtis distances (1/4 root-transformed data) was performed using Past 3.13 (http://folk.uio.no/ohammer/past/).

### Statistical analysis

Statistical analysis was performed using Sigma Plot 12.3 (Systat Software Inc., Carlsbad, CA). Normality was first tested using the Shapiro-Wilk method and equal variance was tested using the Brown-Forsyth method. Depending on normality, *t*-test or non-parametric Mann-Whitney rank sum test was used to test for differences between baseline and post-administration in coverage, richness (number of distinct sequences and Chao1 index), α-diversity (Shannon and Simpson indices), and relative abundance of all operational taxonomic units (OTUs) detected at greater than 0.5% of samples from any one sample site. Prior to testing of all metrics except coverage, data were rarefied via a reiterative random subsampling (10×) of data to the lowest read count obtained for any sample from that site (both time-points included). Mean values from subsampling iterations were then used for testing. Fisher's exact test was used to compare the number of cats in which the probiotic strains were present at week 4 compared to baseline. Testing via PERMANOVA, using Past 3.13, was performed to identify differences in β-diversity between time points and samples sites. Results were considered statistically significant for *p*-values ≤ 0.05.

## Results

**Figure 1:**
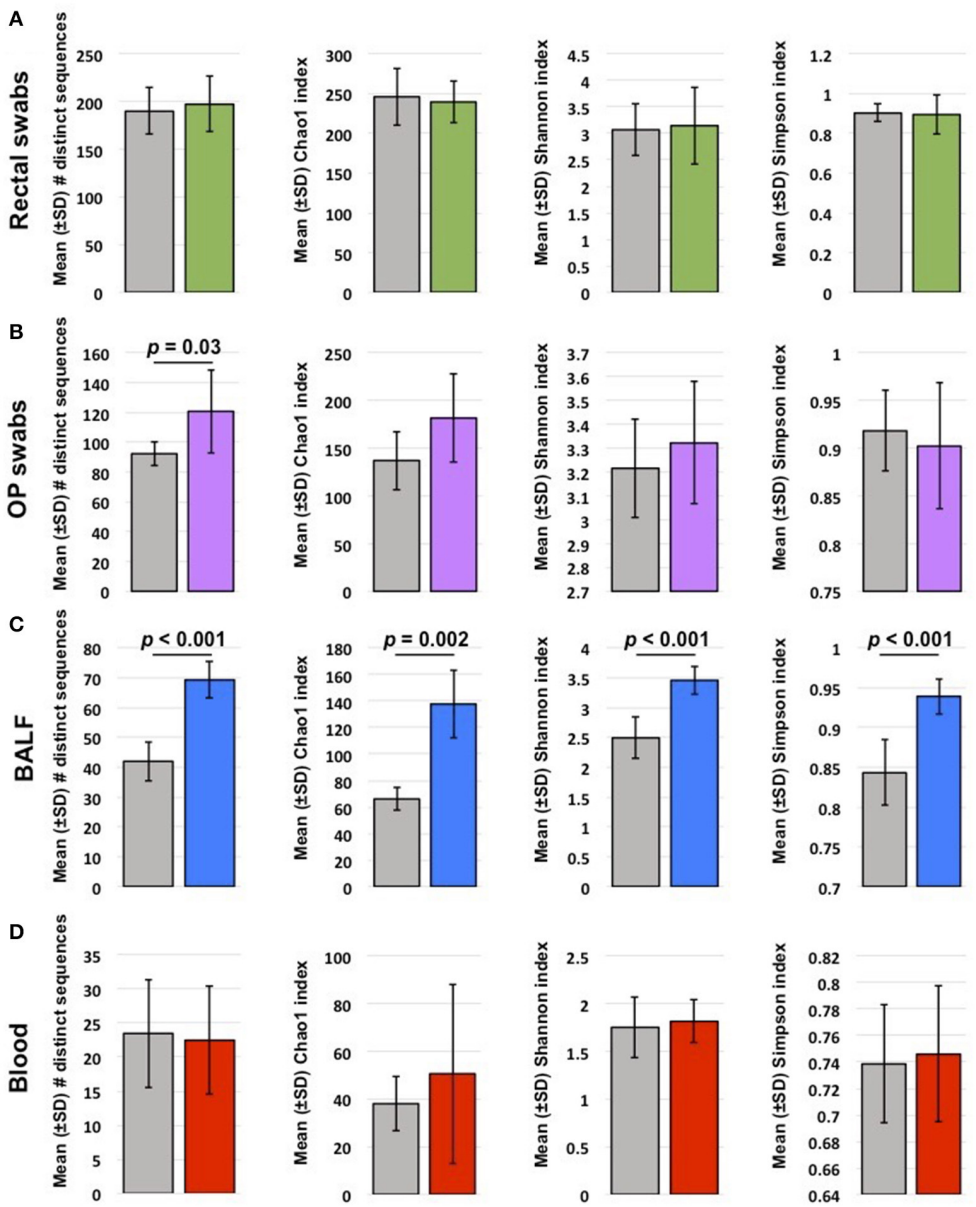
Mean ± standard deviation (SD) richness and diversity of rectal swabs **(A)**, oropharyngeal (OP) swabs **(B)**, bronchoalveolar lavage fluid (BALF) **(C)**, and blood samples **(D)** collected from 6 healthy young cats at baseline (gray bars) and after probiotic administration (colored bars). Richness is represented by the number of distinct sequences detected and Chao1 index; α-diversity is represented by Shannon and Simpson indices. Significant (*p* < 0.05) differences noted above bars (*t*-test or Mann-Whitney rank sum test based on normality).

Coverage (total number of sequences detected) varied by site. Pre-treatment, rectal and OP samples yielded the highest coverage (mean ± SEM of 73,260 ± 8,105 and 36,213 ± 80,395 sequences/sample, respectively), with no significant difference in coverage after probiotic administration (*p* = 0.59; *p* = 0.54, respectively). BALF and blood had lower coverage compared to other sites, presumably due to lower starting microbial biomass. However, the primary comparisons of interest were between baseline and post-VSL3 administration values, rather than between sample sites. Thus, to avoid rarefaction of data from samples such as rectal swabs to the lower read counts obtained from samples such as blood while still accounting for differences in coverage within sample sites, sequence data were rarefied independently for each sample site to the lowest read count obtained for any sample from that sample site. Overall, there was a pattern of decreasing richness (represented by mean number of number of distinct sequences and Chao1 index) progressing from rectal swabs to OP swabs to BALF to blood (Figure 1). Testing for differences in richness within each site revealed significant increases following probiotic administration in samples from OP and BALF, but not blood or rectal swabs (Figure 1). With regard to α-diversity (a metric incorporating both richness and evenness of distribution), time-dependent differences were detected only in BALF samples, with VSL3 administration correlating with increased diversity. Collectively, these data suggest that oral probiotic administration is associated with increased richness and diversity of the airway microbiota, with no concurrent changes detected in the fecal or blood microbiota.

### Sequencing of taxa in the probiotic VSL# 3

Resolved to the taxonomic level of phylum, the probiotic consisted of 59.27% *Actinobacteria* and 40.67% *Firmicutes*. The phylum *Actinobacteria* was predominantly represented by genus *Bifidobacterium* (35.93%) and *Bifidobacterium animalis* (23.29%) in the *Bifidobacteriaceae* family. The phylum *Firmicutes* was represented by genus *Streptococcus* (25.53%) in the *Streptococcaceae* family, and *Lactobacillus zeae* (11.64%) and genus *Lactobacillus* (3.21%) in the *Lactobacillaceae* family.

### Changes in relative abundance of predominant probiotic OTUs varied by site

In rectal samples, *B. animalis* and *L. zeae* increased in relative abundance after probiotic administration (*p* = 0.015 and *p* = 0.004, respectively) but other probiotic OTUs did not change significantly. In OP samples, all probiotic OTUs significantly increased in relative abundance compared to baseline (Table 1). In BALF samples, relative abundance of genus *Streptococcus* (*p* = 0.015), *L. zeae*, (*p* = 0.015), and *B. animalis* (*p* = 0.002) significantly increased. There was no significant difference in relative abundance of the predominant probiotic OTUs in blood samples. The number of cats in which the predominant probiotic OTUs were found in each site at baseline and after probiotic administration is listed in Table 2.

**Table 1:** Relative abundance (mean ± SEM) of the predominant probiotic operational taxonomic units (OTUs) in each site.

**Phylum**	**Family**	**OTU**	**VSL#3**	**Rectal**		**OP**		**BALF**		**Blood**	
				**Baseline**	**Probiotics**	**Baseline**	**Probiotics**	**Baseline**	**Probiotics**	**Baseline**	**Probiotics**
*Actinobacteria*	*Bifidobacteriaceae*	Genus *Bifidobacterium*	35.93	0.02 ± 0.02	0.01 ± 0.00	0.00 ± 0.00	0.05 ± 0.02	0.06 ± 0.06	0.26 ± 0.15	0.00 ± 0.00	0.00 ± 0.00
		*Bifidobacterium animalis*	23.29	0.00 ± 0.00	0.04 ± 0.01	0.00 ± 0.00	0.05 ± 0.01	0.00 ± 0.00	0.35 ± 0.10	0.00 ± 0.00	0.00 ± 0.00
*Firmicutes*	*Lactobacillaceae*	Genus *Lactobacillus*	3.21	0.00 ± 0.00	0.01 ± 0.00	0.00 ± 0.00	0.05 ± 0.02	0.07 ± 0.07	0.05 ± 0.03	0.00 ± 0.00	0.02 ± 0.02
		*Lactobacillus zeae*	11.64	0.00 ± 0.00	0.01 ± 0.00	0.00 ± 0.00	0.15 ± 0.07	0.00 ± 0.00	0.51 ± 0.22	0.00 ± 0.00	0.01 ± 0.01
	*Streptococcaceae*	Genus *Streptococcus*	25.53	4.90 ± 2.19	2.47 ± 1.10	0.20 ± 0.05	0.80 ± 0.20	0.47 ± 0.25	3.31 ± 1.05	0.29 ± 0.19	0.06 ± 0.02

*Shaded cells indicate significant (p < 0.05) increase in relative abundance compared to baseline as determined via Mann-Whitney test. OP, oropharyngeal swab; BALF, bronchoalveolar lavage fluid*.

**Table 2:** Number of cats (out of 6) in which the predominant probiotic operational taxonomic units (OTUs) were sequenced at baseline and after probiotic administration.

**OTU**	**Rectal**		**OP**		**BALF**		**Blood**	
	**Baseline**	**Probiotics**	**Baseline**	**Probiotics**	**Baseline**	**Probiotics**	**Baseline**	**Probiotics**
Genus *Bifidobacterium*	1	5	0	6	1	4	0	0
*Bifidobacterium animalis*	0	5	0	6	0	6	0	0
Genus *Lactobacillus*	4	4	3	6	1	2	0	1
*Lactobacillus zeae*	0	5	0	6	0	5	0	1
Genus *Streptococcus*	6	5	6	6	3	6	2	4

*Shaded cells indicate significant (p < 0.05) increase in detected prevalence compared to baseline as determined via Fisher's exact test. OP, oropharyngeal swab; BALF, bronchoalveolar lavage fluid*.

In addition to introducing some of the predominant probiotic OTUs, probiotic administration was associated with a change in the overall composition of the microbial communities at each site. All taxa that significantly changed after probiotic administration and were detected at greater than 0.50% mean relative abundance in at least one sample site are listed in Supplementary Table 1. The most significant changes in rectal samples included a significant decrease in *Bacteroides ovatus* (mean ± SEM relative abundance of 8.96± 3.50% to 0%; *p* = 0.004), and concurrent increase in *Bacteroides fragilis* (from 2.37 ± 0.86% to 14.31 ± 7.82%; *p* = 0.009), among others. In the upper airways, the following OTUs in the phylum *Proteobacteria* were no longer detected after probiotic administration: *Lautropia* sp. (from 0.95 ± 0.19 to 0%; *p* = 0.004), *Acinetobacter johnsonii* (from 0.02 ± 0.00%; *p* = 0.004) and unclassified (UC) *Moraxellaceae* family (from 1.59 ± 0.31 to 0%; *p* = 0.004). Conversely, there was a significant increase in relative abundance in *Porphyromonas endodontalis* (from 2.47 ± 0.39 to 4.67 ± 0.47%; *p* = 0.004).

Of all samples, the lower airway microbiota underwent the most drastic changes after administration of probiotics. There was a significant decrease in UC *Sphingobacteriaceae* family (from 47.15 ± 5.48 to 13.09 ± 5.69%; *p* = 0.026) and *A. johnsonii* (from 0.85 ± 0.24 to 0 ± 0%; *p* = 0.002). The relative abundance of UC *Sphingobacteriaceae* also significantly decreased in blood samples (from 64.25 ± 2.38 to 33.02 ± 5.20%; *p* = 0.002).

The relative abundance of *Acinetobacter* sp. significantly increased in OP (from 0 ± 0 to 0.17 ± 0.08%; *p* = 0.004), BALF (from 0.07 ± 0.07 to 0.66 ± 0.22%; *p* = 0.015), and blood (from 0 ± 0 to 17.67 ± 8.92%; *p* = 0.015), but not in rectal swabs. This was the only OTU that changed significantly in more than two sites. Larger percentage changes in relative abundance observed in blood samples, and to a lesser extent BALF, are likely a reflection of the lower biomasses in these sites.

### Oral probiotics significantly changed microbial community composition in the lower airways

**Figure 2:**
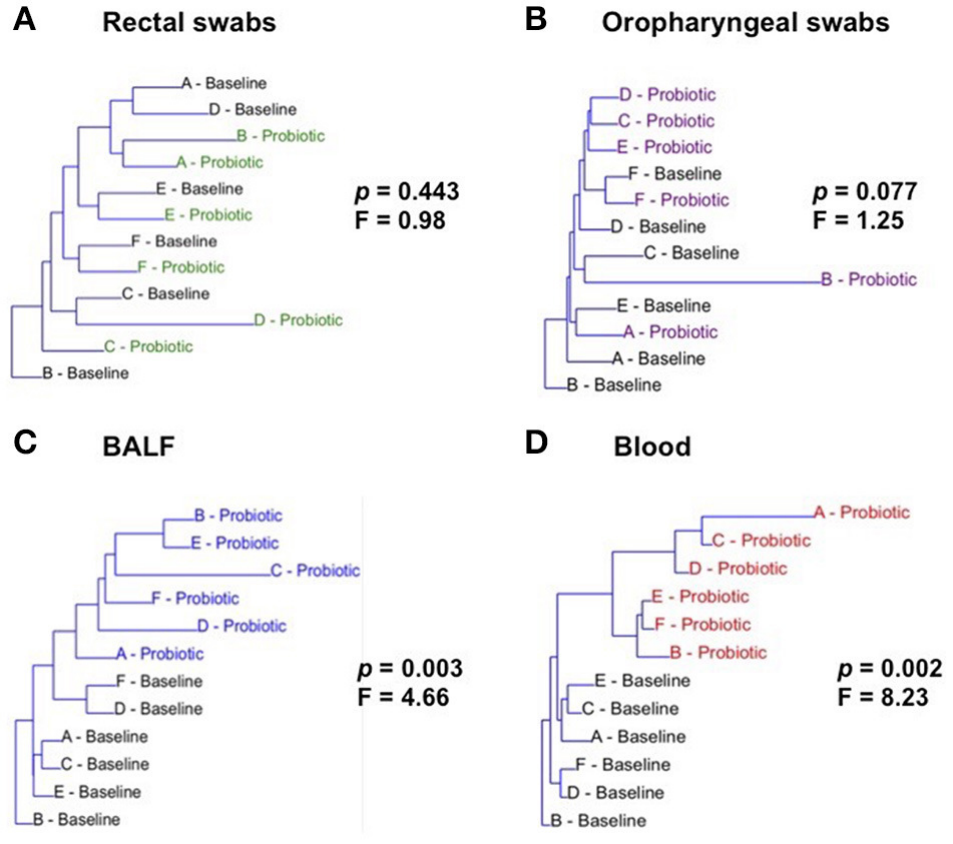
Hierarchical clustering of samples at baseline and after treatment (Probiotic) for all sites: rectal swabs **(A)**, oropharyngeal swabs (OP) **(B)**, bronchoalveolar lavage fluid (BALF) **(C)**, and blood **(D)**. Letters A through F represent each of the cats. *p* and F values determined via one way PERMANOVA of Bray-Curtis distances.

Neighbor joining, hierarchical clustering dendograms were used to visualize the compositional dissimilarity between populations. Regardless of the individual, after treatment, BALF and blood samples were more similar to each other than they were at their corresponding baseline, and were therefore grouped together in the dendogram. In contrast, rectal and OP samples had varied composition among samples independent of treatment (Figure 2). PERMANOVA was used to test for differences in community structure at baseline and after probiotic administration. There were significant differences in microbial community composition in lower airway (*p* = 0.0028; *F* = 4.655) and blood samples (*p* = 0.0022; *F* = 8.233). Interestingly, there was no significant difference in community composition in rectal (*p* = 0.4431; *F* = 0.9843) or OP swabs (*p* = 0.0765; *F* = 1.25) after probiotic administration.

**Figure 3:**
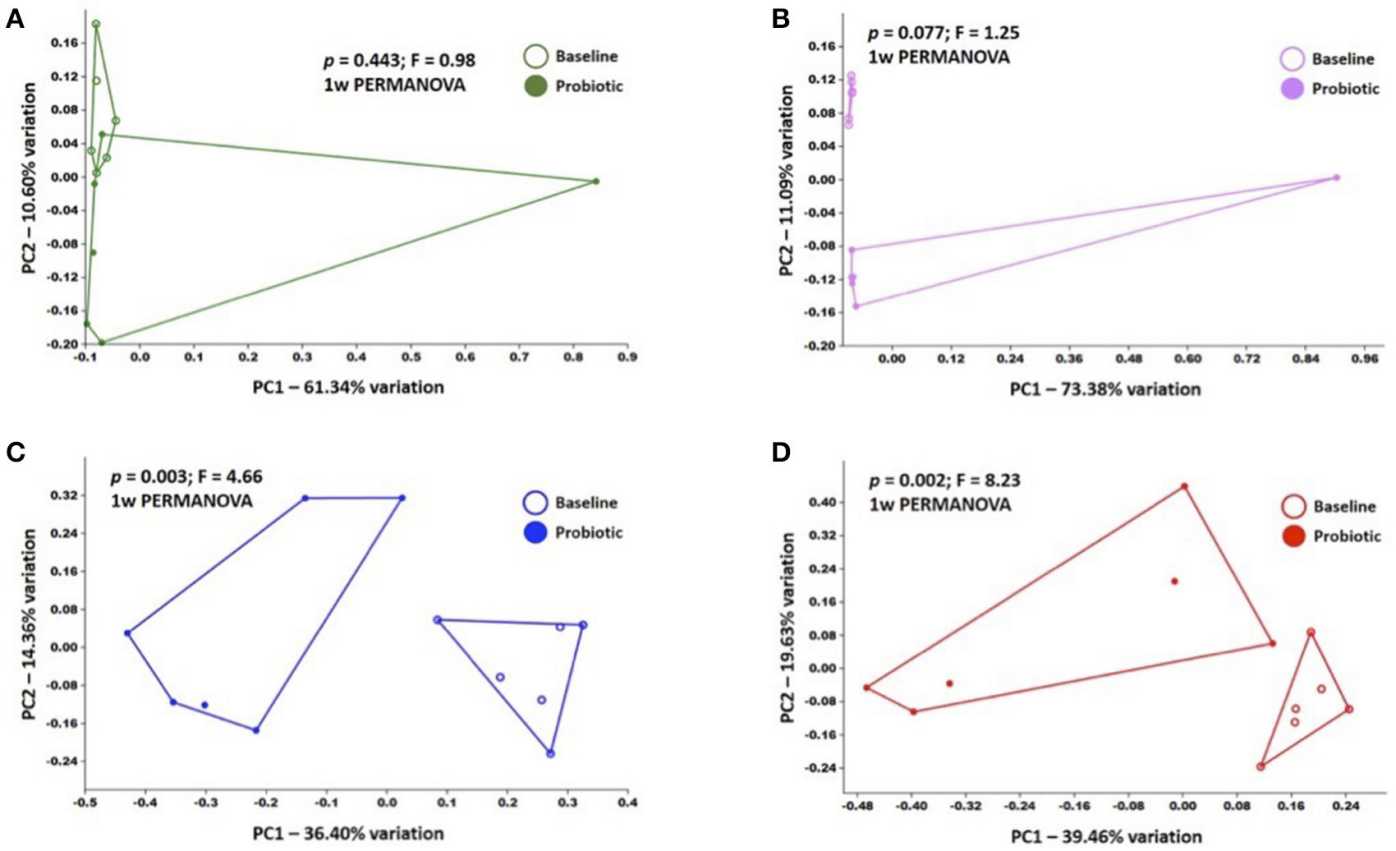
Principal coordinate analyses of baseline and post-administration (Probiotic) samples within each sample site, including rectal swabs **(A)**, OP swabs **(B)**, bronchoalveolar lavage fluid (BALF) **(C)**, and blood **(D)**; legends at right of each plot. *p* and F values determined via one way PERMANOVA of Bray-Curtis distances.

In addition, principal coordinate analysis (PCA) was used to assess the β-diversity of the microbial communities found at all sample sites collectively and at each site independently. When samples from all sites at baseline and after probiotic administration were included in the analysis, principal coordinates 1 and 2 (PC1 and PC2), accounting for 34.38 and 19.52% of the variation respectively, showed complete separation of the rectal, OP, and blood bacterial populations while BALF communities overlapped all of the other sites (Supplementary Figure 1). Of note, there was one rectal swab sample and one OP swab sample, both collected post probiotic administration that demonstrated a markedly different composition than the others in the group. These samples were not from the same cat and the reason for this divergence is unknown. Testing for main effects via two-way PERMANOVA found significant main effects of both sample site (*p* = 0.0001; *F* = 22.05) and time-point (*p* = 0.0041; *F* = 4.31), as well as a significant interaction (*p* = 0.01; *F* = 2.25). Recognizing that the inherent and well-known compositional differences between microbial communities present in the sites tested could obscure time-dependent differences within each sample site, we also performed PCA of samples from each site independently (Figure 3). Confirming what was seen in the hierarchical clustering analysis, there was complete separation of baseline and post-probiotic BALF and blood samples along PC1. The discordant post-administration samples within the rectal swab and OP swab sites skewed the PCA plots and any apparent separation of pre- and post-probiotic samples within those sample sites occurred along PC2 (accounting for a modest 10.6 and 11.1% variation respectively) and did not achieve significance. Collectively, these analyses suggest that while there were negligible, if any, probiotic-dependent effects on the composition of the rectal or OP microbial communities, oral VSL3 administration was associated with significant compositional changes in the microbial DNA detected in the BALF and blood. Taking these data in the context of the richness and α-diversity data reported above, we believe these findings to support the hypothesis that oral administration of certain probiotic species can significantly alter the resident airway microbiota, both in overall composition and richness and diversity.

## Discussion

This is the first study to document that in the healthy cat, orally administered probiotics have the potential to change microbial communities at sites distant from the gastrointestinal (GI) tract. Traditionally, probiotics have been employed as a way to modulate the GI microbiota to promote local health (Lievin-Le Moal and Servin, 2014). In conjunction with the resident microbiota, probiotics can help inhibit colonization and/or proliferation of pathogens by interfering with adhesion to the intestinal mucosa and outcompeting pathogens for shared nutrients (Guarner et al., 2012). There is growing evidence that oral probiotics not only influence the GI tract directly, but also have immunomodulatory effects at distant sites, including the urogenital and respiratory tracts, oral cavity/dentition, mammary gland and skin. Although oral probiotics can prevent and/or decrease frequency or severity of diseases outside of the gastrointestinal tract (i.e., an indirect effect) (Alberda et al., 2007; Lappin et al., 2009; Di Nardo et al., 2014; Esposito et al., 2014; Lee et al., 2015; Zuccotti et al., 2015; Fuchs-Tarlovsky et al., 2016; Gruner et al., 2016; Vieira et al., 2016; Zamani et al., 2016), few studies have directly evaluated the appearance of orally administered probiotic species or changes in host microbial communities at distant sites (De Alberti et al., 2015; Mastromarino et al., 2015; Treven et al., 2015). A recent study in pre-menopausal women demonstrated an increase in orally administered probiotic species in vaginal swabs using qPCR analysis, setting the stage for use of oral probiotics to prevent or treat urogenital infections (Mezzasalma et al., 2017). A combination of bovine lactoferrin and two probiotics administered to healthy women led to detection of the probiotic species in vaginal swabs, important pilot data for future studies of bacterial vaginosis (De Alberti et al., 2015). Oral probiotics given to mice during pregnancy and lactation modulated the composition of bacterial communities in the mesenteric lymph nodes, mammary gland and milk with implications for neonatal health (Treven et al., 2015). Results were similar in women administered oral probiotics from late pregnancy through lactation showing significant increases in probiotic species in colostrum and milk (Mastromarino et al., 2015). A study relevant to prevention of dental caries employed 16S rRNA pyrosequencing to demonstrate changes in the composition of the microbiome in the oral cavity, specifically tooth biofilms, after 12 weeks of supplementation (Romani Vestman et al., 2015).

Probiotics have appeal, as there is growing evidence that they can improve health in patients with dysbiosis. The existence of a gut-lung axis allowing cross-talk through the CMIS has been proposed as a potential explanation for how microbes in the GI tract might influence immune functions in the lungs relevant in health and disease (Marsland et al., 2015; He et al., 2017). One potential mechanism is through interactions between host cell pattern recognition receptors (PRRs) and gut-derived pathogen-associated molecular patterns (PAMPs) leading to innate immune responses as well as systemic and CMIS immunoregulation (Neish, 2014; Davies and Abreu, 2015). Another mechanism is through production of bacterial metabolites such as short-chain fatty acids, which provide broad local and systemic immune effects (Meijer et al., 2010; Marsland and Salami, 2015; Budden et al., 2016). Additionally, dendritic cells that reside in the GI lamina propria or gut associated lymphoid tissue can sample members of the gut microbiota; interactions allow for maturation and migration of DCs to draining lymph nodes key for homeostatic adaptive immune responses (Samuelson et al., 2015). DCs activate various T cells subsets, which in turn are imprinted with chemokine receptors allowing homing to distal mucosal sites such as the respiratory tract (Samuelson et al., 2015). In addition to the gut contributing to immune homeostasis and immune responses in the lung, there is bi-directional exposure of microbiota at each site: inhaled microbes (as demonstrated by nasal cavity deposition in mice) consistently appear in the GI tract, and microaspiration of GI microbes is an important route by which the respiratory microbiota is established (Southam et al., 2002; Beigelman et al., 2014; Dickson and Huffnagle, 2015; Adar et al., 2016).

The mechanisms of action by which oral probiotics modify differing populations of microbial communities at various body sites and/or influence the host immune system are multifactorial and not fully understood. Our study supports the appearance of probiotic bacterial species in the upper and lower airways that were absent or in decreased abundance prior to the probiotic trial. We speculate that clinically silent microaspiration may have contributed to seeding of probiotic species in the respiratory tract. Compositional similarities between the blood and lower airway microbiome bring into question the possibility of GI tract bacterial translocation from the gut through the bloodstream as a route of entry; however, the bacterial communities in the GI tract were significantly different from those in blood and BAL. Perhaps as has been suggested in other studies, a systemic effect of oral probiotics (e.g., alteration of cytokine concentrations) may have modulated the respiratory microbiota (Vitali et al., 2012; Dai et al., 2013; Hepburn et al., 2013; Mastromarino et al., 2015).

In line with multiple other studies (Madan et al., 2012; Morris et al., 2013; Budden et al., 2016; Dickson et al., 2016) and as mentioned above, our results support microaspiration as a primary route of microbial colonization of the lower airways. In the current study, all of the predominant probiotic OTUs significantly increased in relative abundance in the upper airways, genus *Streptococcus* increased in the lower airways and *L. zeae*, and *B. animalis* increased in the lower airways and GI tract post-treatment. In healthy individuals, the respiratory microbiota is postulated to be established by a balance between entry (inhalation, microaspiration) and removal (e.g., via the mucociliary apparatus) of microbes and unlike the gut, there is little evidence for long-term colonization from local microbial reproduction (Dickson et al., 2016). This, along with the lower biomass, may explain why the probiotic OTUs more significantly altered the lower airways compared with the GI tract.

Several studies have reported benefits of probiotics to lung health (Forsythe, 2011, 2014; Yoda et al., 2012). Understanding the influence of oral probiotics on distant respiratory tract microbiota is crucial for future studies evaluating therapeutic applications in respiratory diseases like allergic asthma that affect humans and cats. Spontaneous and experimentally induced feline asthma shares the hallmark features of human allergic asthma, such as airway eosinophilia, airway hyper-responsiveness, and airway remodeling, making cats a valuable translational model for this disease (Reinero et al., 2009; Williams and Roman, 2016). Allergic asthma results from stimulation and amplification of a local inflammatory response mediated by allergen-specific T helper 2 (T_H_2) cells that occurs in genetically susceptible individuals exposed to environmental triggers. Recent studies in humans have provided evidence that dysbiosis occurs in asthma (Hilty et al., 2010; Goleva et al., 2013; Huang, 2013; Gollwitzer and Marsland, 2014).

The original hygiene hypothesis stated that improved hygiene and reduced exposure of the immune system to microbial stimulus during infancy and early childhood predisposes to impaired immunoregulation and an increase in prevalence of allergic diseases (Beigelman et al., 2014). Allergic diseases are driven by T_H_2 cells and many childhood infections trigger a T_H_1 immune response, therefore by having fewer childhood infections the immune system is thought to be polarized toward T_H_2 immune responses, promoting allergies. However, allergic diseases are not the only type of inflammatory diseases that have been on the rise; other T_H_1-driven chronic inflammatory responses, such as inflammatory bowel disease, have been increasing in developed countries (Haahtela et al., 2013; McCoy and Koller, 2015). This has led to the evolution of the hygiene hypothesis to the biodiversity or the microbiota hypothesis. The microbiota hypothesis proposes that dysbiosis or disruption of the host commensal microbiota disrupts the relationship between the microbes and the immune system, leading to immune dysfunction (Haahtela et al., 2013).

Dysbiosis leads to multiple important consequences: overgrowth of pathogens that compete with commensal microbes for host binding sites, loss of commensal microbial diversity, and a host inflammatory response that contributes to disease development (Fujimura et al., 2010). Chronic inflammation further disrupts the microbiota leading to a self-perpetuating inflammatory state. Return of the microbiota to a normal/healthy state has appeal in treatment of various disorders and studies have shown, for instance, that therapeutic alteration of the GI microbiota can promote remission of disease. Fecal microbiota transplantation is a classic example in which microbiota from healthy individuals are transplanted into patients with *Clostridium difficile* overgrowth, thereby inducing disease remission (Borody et al., 2004). Several studies in murine models of allergic airway disease indicate that probiotics can promote a tolerogenic environment in the lungs via induction of CD4^+^CD25^+^FoxP3^+^ T regulatory cells (Feleszko et al., 2007; Karimi et al., 2009), altering T_H_2 cytokine profiles (Forsythe et al., 2007; Fujimura et al., 2014) and modulating allergen-specific antibody production (Alvarez et al., 2001). In addition, multiple studies in experimental asthma models using oral probiotics have reported reduction of the asthmatic phenotype including airway inflammation and airflow limitation (Forsythe et al., 2007; Inoue et al., 2009; Karimi et al., 2009; Hougee et al., 2010; Li et al., 2010).

This is the first study to evaluate the effect of orally administered probiotics on the microbial communities in the airways and blood by determining changes in relative abundance of predominant probiotic OTUs. These data support microaspiration being one of the routes for lower airway microbial colonization and seeding of probiotic species. After oral administration, not only were the predominant probiotic OTUs present in distant sites, but they were also associated with changes in richness and the overall composition of colonizing microbial populations of the respiratory tract and blood.

We acknowledge there was a significant difference in coverage and richness at baseline compared to after treatment. Aside from this being secondary to probiotic administration, it could be argued that maturation of the cats may play a role in both richness and coverage, and this differential coverage could lead to changes in richness. However, we would argue that the changes in coverage and richness do not influence whether or not a particular OTU would be detected in a sample, and would speculate that this change is secondary to probiotic administration. These data support the idea that orally administered probiotics could be suited for modulation of upper and lower airway microbiota diseases relevant to both human and veterinary medicine.

## Availability of supporting data

All sequence data have been deposited in the NCBI Short Read Archive (SRA) under the accession number PRJNA376735.

## Author contributions

AV participated in the conception and design of the study, sample collection, DNA extraction, data interpretation and drafted manuscript. AE participated in the conception and design of the study, interpreted sequence data and helped to draft the manuscript. CR participated in sample collection, the conception and design of the study and data interpretation, and helped to draft the manuscript. HR assisted with DNA extraction, sample collection, and study coordination. All authors read and approved the final manuscript.

## Authors information

AVP, resident in the MU College of Veterinary Medicine (CVM) Department of Veterinary Medicine and Surgery, is a veterinarian currently completing specialty training in small animal internal medicine. AE, Research Assistant Professor in the MU CVM Department of Veterinary Pathobiology, is a veterinarian with specialty training in laboratory animal and comparative medicine, and Director of the University of Missouri Metagenomics Center. CR, Associate Professor in the MU CVM Department of Veterinary Medicine and Surgery, is a veterinarian with specialty training in small animal internal medicine, and Director of the MU Comparative Internal Medicine Laboratory. HR is a Senior Research Associate in the Comparative Internal Medicine Laboratory.

### Conflict of interest statement

The authors declare that the research was conducted in the absence of any commercial or financial relationships that could be construed as a potential conflict of interest.

## Supplementary material

Supplementary Figure 1 β-diversity as shown via principal coordinate analysis of Bray-Curtis distances between samples from all four sample sites (rectal swab, OP swab, BALF, and blood); legend at right.

Supplementary Table 1 Relative abundance of all taxa detected at greater than 0.50% mean relative abundance in at least one sample site (feces, oropharyngeal swabs (OP), bronchoalveolar lavage (BAL) or blood), that changed significantly after probiotic administration, annotated to the level of phylum, family, and operational taxonomic unit (OTU). Data presented as mean ± standard error of the mean (SEM). ^*^Shaded cells indicate *p* = <0.05 compared to baseline.

## Abbreviations

BALF: bronchoalveolar lavage fluid
CMIS: common mucosal immune system
GI: gastrointestinal
OP: oropharyngeal
OTU: operational taxonomic unit
UC: unclassified
PCA: principal component analysis
T_H_: T helper.
