# Supplementary figures and images for: Oral Probiotics Alter Healthy Feline Respiratory Microbiota
Source: Front Microbiol. 2017 Jul 11;8:1287. doi: 10.3389/fmicb.2017.01287 (PMC5504723; doi:10.3389/fmicb.2017.01287)

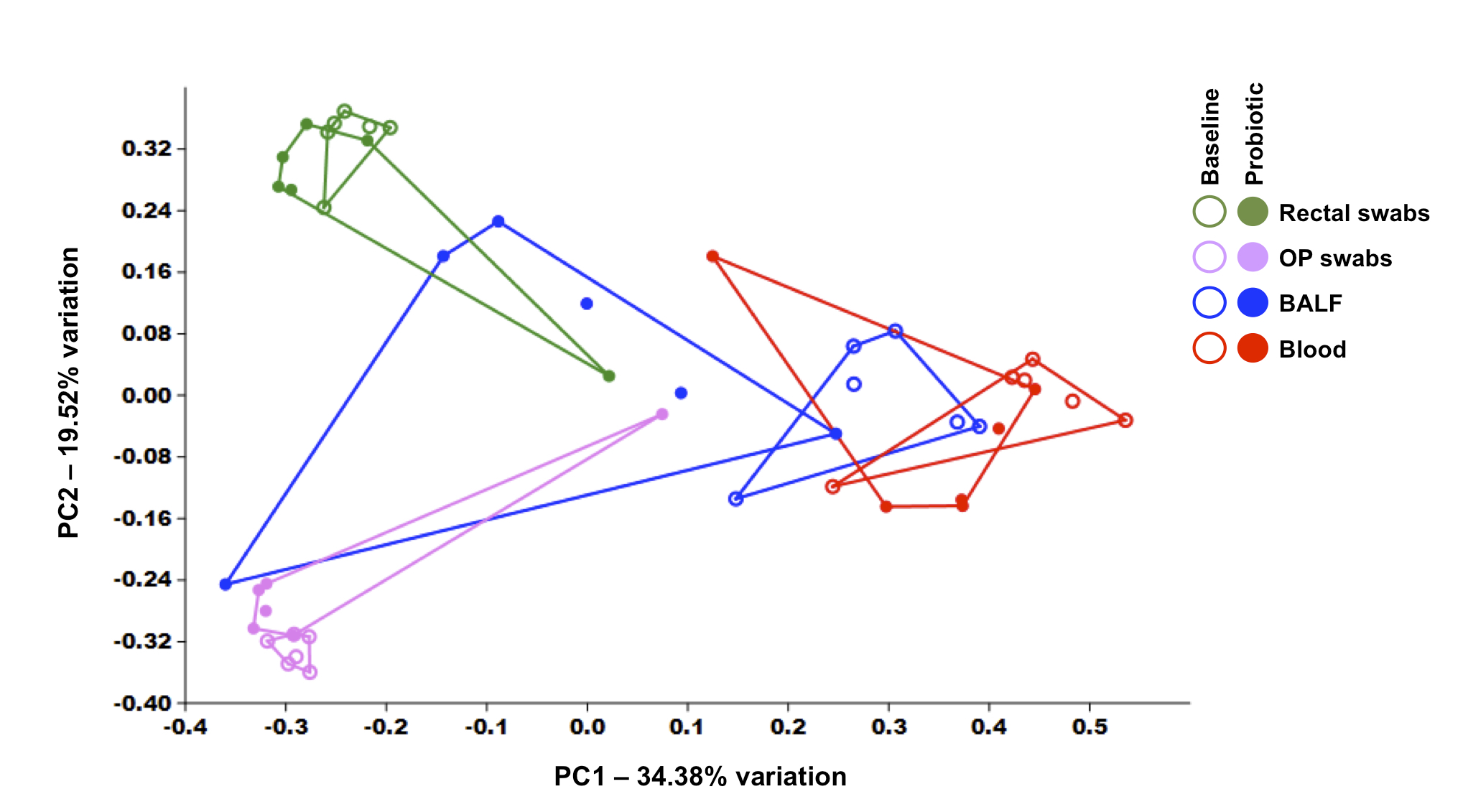

Supplement: Supplementary Figure 1 — β-diversity as shown via principal coordinate analysis of Bray-Curtis distances between samples from all four sample sites (rectal swab, OP swab, BALF, and blood); legend at right. [file Image1.JPEG]
