# Supplementary material for: Oral Probiotics Alter Healthy Feline Respiratory Microbiota
Source: Front Microbiol. 2017 Jul 11;8:1287. doi: 10.3389/fmicb.2017.01287 (PMC5504723; doi:10.3389/fmicb.2017.01287)
Supplement: Supplementary Table 1 — Relative abundance of all taxa detected at greater than 0.50% mean relative abundance in at least one sample site (feces, oropharyngeal swabs (OP), bronchoalveolar lavage (BAL) or blood), that changed significantly after probiotic administration, annotated to the level of phylum, family, and operational taxonomic unit (OTU). Data presented as mean ± standard error of the mean (SEM). *Shaded cells indicate p = <0.05 compared to baseline. [file Table1.PDF]

Supplementary Table 1. Relative abundance of all taxa detected at greater than 0.50% mean relative abundance in at least one sample site (feces, oropharyngeal swabs (OP), bronchoalveolar lavage (BAL) or blood), that changed significantly after probiotic administration, annotated to the level of phylum, family, and operational taxonomic unit (OTU). Data presented as mean ± standard error of the mean (SEM). *Shaded cells indicate $p = < 0.05$ compared to baseline.

Phylum	Family	O T U	Rectal		OP		BALF		Blood	
			Baseline	Probiotic	Baseline	Probiotic	Baseline	Probiotic	Baseline	Probiotic
Actinobacteria	*Corynebacteriaceae*	*Corynebacterium sp.*	1.48 ± 0.56	1.72 ± 1.07	0.01 ± 0.00	0.05 ± 0.03	0.12 ± 0.08	0.38 ± 0.17	0.00 ± 0.00	0.04 ± 0.04
	Propionibacteriaceae	*Propionibacterium acnes*	0.00 ± 0.00	0.02 ± 0.01	0.07 ± 0.05	0.18 ± 0.06	0.33 ± 0.27	3.33 ± 1.28	0.13 ± 0.08	0.10 ± 0.04
Bacteroidetes	*Bacteroidaceae*	*Bacteroides fragilis*	2.37 ± 0.86	14.31 ± 7.82	0.00 ± 0.00	0.03 ± 0.01	0.19 ± 0.14	2.51 ± 1.17	0.00 ± 0.00	0.13 ± 0.08
		Bacteroides ovatus	8.96 ± 3.50	0.00 ± 0.00	0.01 ± 0.00	0.00 ± 0.00	0.45 ± 0.16	0.00 ± 0.00	0.17 ± 0.07	0.00 ± 0.00
	Flavobacteriaceae	*Capnocytophaga canimorsus*	0.00 ± 0.00	0.00 ± 0.00	0.57 ± 0.08	0.00 ± 0.00	0.07 ± 0.07	0.00 ± 0.00	0.05 ± 0.05	0.00 ± 0.00
	Porphyromonadaceae	*Porphyromonas endodontalis*	0.00 ± 0.00	0.00 ± 0.00	2.47 ± 0.39	4.67 ± 0.47	0.06 ± 0.06	0.67 ± 0.56	0.00 ± 0.00	0.00 ± 0.00
	Rikenellaceae	*Rikenellaceae sp.*	0.01 ± 0.00	0.00 ± 0.00	0.00 ± 0.00	0.00 ± 0.00	0.78 ± 0.51	0.00 ± 0.00	0.75 ± 0.52	0.00 ± 0.00
	S24-7	Family *S24-7*	0.07 ± 0.02	0.07 ± 0.06	0.01 ± 0.00	0.00 ± 0.00	1.01 ± 0.28	0.03 ± 0.03	0.79 ± 0.32	0.01 ± 0.01
	Sphingobacteriaceae	Family *Sphingobacteriaceae*	0.02 ± 0.02	0.00 ± 0.00	0.00 ± 0.00	0.00 ± 0.00	47.15 ± 5.48	13.09 ± 5.69	64.25 ± 2.38	33.02 ± 5.20
Firmicutes	*[Tissierellaceae]*	*Anaerococcus sp.*	2.80 ± 1.02	2.15 ± 1.23	0.01 ± 0.00	0.02 ± 0.01	0.05 ± 0.05	1.50 ± 0.72	0.00 ± 0.00	0.18 ± 0.18
	Lachnospiraceae	*[Ruminococcus] gnavus*	0.44 ± 0.12	0.53 ± 0.21	0.00 ± 0.00	0.01 ± 0.01	0.00 ± 0.00	0.30 ± 0.19	0.00 ± 0.00	0.00 ± 0.00
		[Ruminococcus] sp.	0.69 ± 0.27	0.56 ± 0.32	0.00 ± 0.00	0.00 ± 0.00	0.00 ± 0.00	0.15 ± 0.05	0.00 ± 0.00	0.01 ± 0.01
		Blautia sp.	0.56 ± 0.17	0.40 ± 0.17	0.00 ± 0.00	0.01 ± 0.00	0.00 ± 0.00	0.18 ± 0.06	0.00 ± 0.00	0.00 ± 0.00
		Roseburia sp.	5.14 ± 1.92	7.16 ± 3.85	0.00 ± 0.00	0.03 ± 0.02	0.00 ± 0.00	0.55 ± 0.28	0.00 ± 0.00	0.00 ± 0.00
	Order *Clostridiales*	Order *Clostridiales*	0.75 ± 0.32	0.88 ± 0.71	0.12 ± 0.04	0.13 ± 0.04	0.94 ± 0.36	0.21 ± 0.08	0.70 ± 0.53	0.07 ± 0.04
	Ruminococcaceae	*Oscillospira sp.*	0.60 ± 0.06	0.41 ± 0.14	0.00 ± 0.00	0.01 ± 0.00	0.39 ± 0.20	0.11 ± 0.05	0.47 ± 0.17	0.02 ± 0.02
	Staphylococcaceae	*Staphylococcus sp.*	1.32 ± 0.70	1.45 ± 0.91	0.02 ± 0.00	0.10 ± 0.02	0.31 ± 0.20	2.56 ± 1.03	0.30 ± 0.10	0.01 ± 0.01
Proteobacteria	*Burkholderiaceae*	*Lautropia sp.*	0.01 ± 0.01	0.00 ± 0.00	0.95 ± 0.19	0.00 ± 0.00	0.00 ± 0.00	0.00 ± 0.00	0.00 ± 0.00	0.00 ± 0.00
	Moraxellaceae	*Acinetobacter johnsonii*	0.01 ± 0.00	0.00 ± 0.00	0.02 ± 0.01	0.00 ± 0.00	0.85 ± 0.24	0.00 ± 0.00	0.25 ± 0.12	0.00 ± 0.00
		Family *Moraxellaceae*	0.00 ± 0.00	0.00 ± 0.00	1.59 ± 0.31	0.00 ± 0.00	0.00 ± 0.00	0.00 ± 0.00	0.00 ± 0.00	0.00 ± 0.00
		Acinetobacter sp.	0.00 ± 0.00	0.02 ± 0.02	0.00 ± 0.00	0.17 ± 0.08	0.07 ± 0.07	0.66 ± 0.22	0.00 ± 0.00	17.67 ± 8.92
	Pasteurellaceae	Family *Pasteurellaceae*	0.01 ± 0.01	0.01 ± 0.01	10.51 ± 3.70	17.25 ± 4.53	0.29 ± 0.29	3.18 ± 2.20	0.05 ± 0.05	0.01 ± 0.01
